# Single-cell transcriptomic analysis of renal allograft rejection reveals insights into intragraft TCR clonality

**DOI:** 10.1172/JCI170191

**Published:** 2023-07-17

**Authors:** Tiffany Shi, Ashley R. Burg, J. Timothy Caldwell, Krishna M. Roskin, Cyd M. Castro-Rojas, P. Chukwunalu Chukwuma, George I. Gray, Sara G. Foote, Jesus A. Alonso, Carla M. Cuda, David A. Allman, James S. Rush, Catherine H. Regnier, Grazyna Wieczorek, Rita R. Alloway, Adele R. Shields, Brian M. Baker, E. Steve Woodle, David A. Hildeman

**Affiliations:** 1 Division of Immunobiology and; 2 Immunology Graduate Program, Cincinnati Children’s Hospital Medical Center, Cincinnati, Ohio, USA.; 3 Medical Scientist Training Program and; 4 Division of Transplantation, Department of Surgery, University of Cincinnati College of Medicine, Cincinnati, Ohio, USA.; 5 Division of Nephrology and Hypertension and; 6 Divison of Biomedical Informatics, Cincinnati Children’s Hospital Medical Center, Cincinnati, Ohio, USA.; 7 Department of Chemistry and Biochemistry and the Harper Cancer Research Institute, University of Notre Dame, Notre Dame, Indiana, USA.; 8 Northwestern University, Feinberg School of Medicine, Department of Medicine, Division of Rheumatology, Chicago, Illinois, USA.; 9 University of Pennsylvania Perelman School of Medicine, Philadelphia, Pennsylvania, USA.; 10 Novartis Institutes for Biomedical Research, Immunology Disease Area, Basel, Switzerland.; 11 Division of Nephrology and Hypertension, Department of Internal Medicine, and; 12 Department of Pediatrics, University of Cincinnati College of Medicine, Cincinnati, Ohio, USA.

**Keywords:** Immunology, Transplantation, Organ transplantation, T cell receptor, T cells

## Abstract

Bulk analysis of renal allograft biopsies (rBx) identified RNA transcripts associated with acute cellular rejection (ACR); however, these lacked cellular context critical to mechanistic understanding of how rejection occurs despite immunosuppression (IS). We performed combined single-cell RNA transcriptomic and TCR-α/β sequencing on rBx from patients with ACR under differing IS drugs: tacrolimus, iscalimab, and belatacept. We found distinct CD8^+^ T cell phenotypes (e.g., effector, memory, exhausted) depending upon IS type, particularly within expanded CD8^+^ T cell clonotypes (CD8_EXP_). Gene expression of CD8_EXP_ identified therapeutic targets that were influenced by IS type. TCR analysis revealed a highly restricted number of CD8_EXP_, independent of HLA mismatch or IS type. Subcloning of TCR-α/β cDNAs from CD8_EXP_ into Jurkat 76 cells (TCR^–/–^) conferred alloreactivity by mixed lymphocyte reaction. Analysis of sequential rBx samples revealed persistence of CD8_EXP_ that decreased, but were not eliminated, after successful antirejection therapy. In contrast, CD8_EXP_ were maintained in treatment-refractory rejection. Finally, most rBx-derived CD8_EXP_ were also observed in matching urine samples, providing precedent for using urine-derived CD8_EXP_ as a surrogate for those found in the rejecting allograft. Overall, our data define the clonal CD8^+^ T cell response to ACR, paving the next steps for improving detection, assessment, and treatment of rejection.

## Graphical abstract

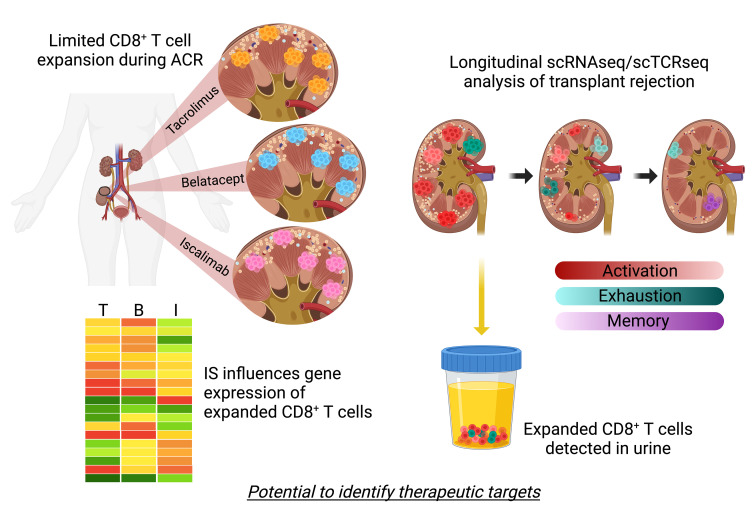

## Introduction

Transplantation is the most effective treatment for kidney failure, providing improved survival and quality of life. To prevent rejection, patients are maintained on lifelong immunosuppression (IS) therapy, the standard-of-care being the calcineurin inhibitor (CNI) tacrolimus. Although current CNI-based regimens provide low acute rejection rates (~10%) in the first posttransplant year, thereafter patients remain at risk of therapeutically resistant late rejection that occurs at a rate of 1%–3% per year. In addition, CNI-based regimens are associated with substantial toxicities due to off-target effects (1, 2). To avoid CNI toxicities, targeted IS agents that block T cell costimulation are being developed. Belatacept (CTLA4-Ig) is FDA approved, and despite increased rejection rates 1 year after transplant, long-term kidney function is increased under belatacept IS (3). However, belatacept-refractory rejection (BRR) episodes are more severe and more difficult to treat than rejection episodes that occur under CNI-based IS (4–6). Antibody-mediated blockade of the CD40/CD40L costimulatory pathway has shown promise as maintenance IS in nonhuman primates (7–9) and in pig-to-monkey xenografts (10–12). Fully human anti-CD40 monoclonal antibodies, such as iscalimab and bleselumab, have provided effective IS in human trials, but remained less efficacious compared with tacrolimus-based treatment in the prevention of organ rejection (13, 14). Thus, although costimulatory blockade approaches are promising, less toxic IS regimens, rejection occurring under these regimens remains poorly understood.

Alloreactive CD8^+^ T cells present a major barrier to allograft acceptance, as they are major drivers of acute cellular rejection (ACR) (15). Insights into graft-infiltrating T cells and their clonality during allograft rejection were revealed with bulk TCR-sequencing analyses in prior studies (16-20). However, these studies only sequenced TCR-β chains, which, in absence of TCR-α chain sequencing, do not indicate true clonality or T cell type (e.g., CD4^+^ or CD8^+^). Similarly, bulk RNA-Seq (bulkseq) has identified rejection-associated transcripts (21–23), which contributed to the understanding of transplant rejection on the transcriptomic level. However, a major limitation of bulkseq analyses (both TCR and whole transcriptome) is that they do not attribute mRNA transcripts to individual cells, particularly T cells driving allograft rejection.

In contrast with bulkseq, single-cell RNA-Seq (scRNA-Seq) has enabled transcriptomic analysis of individual cells (24). Additionally, single-cell TCR sequencing (scTCR-Seq) enables assessment of T cell clonality (by single-cell pairing of TCR-α/β chains) in combination with scRNA-Seq (25). Although scRNA-Seq was used to characterize macrophages in a patient undergoing mixed rejection (26) and to define donor versus recipient leukocytes in patients undergoing antibody-mediated rejection (27), single-cell analysis of transcripts and CD8^+^ T cell clonality in the acutely rejecting renal allograft has not been performed. Another study profiled bronchoalveolar lavage (BAL) fluid cells by scRNA-Seq from patients undergoing ACR of lung allografts before and after treatment with glucocorticoids (28). However, analysis of lung tissue during rejection was not performed, so the relationship of BAL fluid–derived T cells to T cells and other immune cells infiltrating lung allograft tissue during rejection remains unclear.

Here, we provide what we believe is the first combined scRNA-Seq/scTCR-Seq analysis of human kidney allograft biopsies from patients undergoing ACR, including analyses of serial biopsies over time and comparative analyses of paired urine and allograft biopsy samples. Our analyses yield important findings on the nature of human renal allograft rejection, including the following: (a) remarkable restriction of CD8^+^ T cell clonal expansion; (b) type of maintenance IS affecting gene expression within expanded CD8^+^ T cell clonotypes (CD8_EXP_) observed in index biopsies (biopsies obtained at time of rejection); (c) persistence of CD8_EXP_ observed in both index and subsequent renal allograft biopsies (rBx) (even months later), reflecting long-term clonal persistence and adaptation despite rejection treatment; and (d) correlation of CD8_EXP_ observed in renal allograft rejection biopsies with those obtained in urinary sediment. Together, these results provide fundamental insights into allograft rejection and how CD8_EXP_ respond to antirejection therapies. Our results indicate that combined scRNA-Seq/scTCR-Seq has the potential to instruct the personalization and enhancement of antirejection therapy to improve long-term allograft survival.

## Results

### scRNA-Seq analysis of acutely rejecting human kidney allografts.

**Figure 1:**
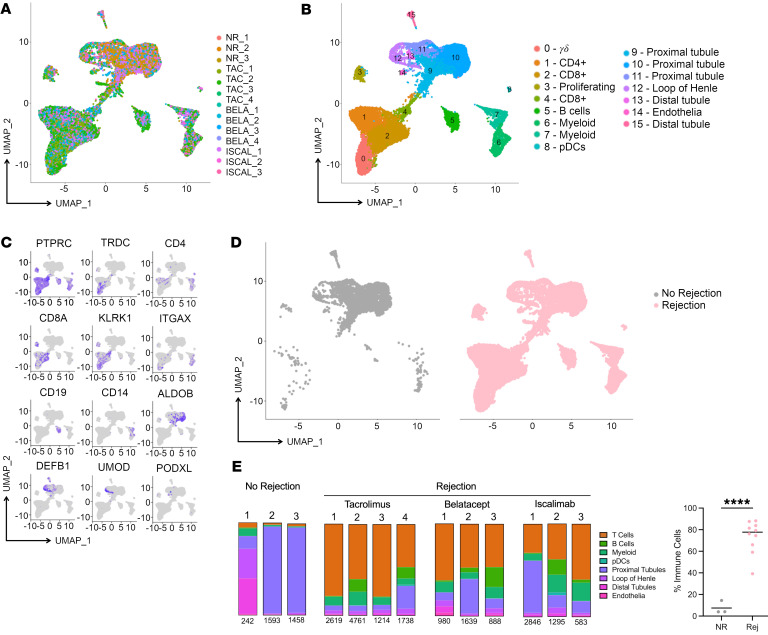
scRNA-Seq analysis of transplanted kidney allografts. Single-cell suspensions from 13 different biopsies (3 without rejection, 10 with rejection) were individually subjected to 5′ scRNA-Seq on the 10× platform with V(D)J sequencing. After alignment using Cell Ranger, cells with more than 25% mitochondrial content and less than 200 genes, including additional low-quality cells, were removed, and samples were integrated using Seurat. (**A** and **B**) UMAP plots display cell contribution by sample and cell type. (**C**) Expression of “signature” genes across cell types. Blue color intensity reflects the expression level of individual genes within given cells. (**D**) Separation of samples based on rejection status. UMAP plots show cells from no-rejection samples (gray, left plot) versus rejection samples (pink, right plot). (**E**) Frequency of cell types within each sample displayed in bar graphs. Statistical analyses reveal a significantly increased proportion of immune infiltration in the rejection samples (*n* = 10) compared with the no-rejection samples (*n* = 3). Two-tailed *t* test, ****P < 0.0001.

To understand ACR at the single-cell level, we performed scRNA-Seq with 5′ V(D)J sequencing on index kidney allograft biopsies obtained from 13 individual participants: 10 biopsies from participants undergoing an ACR episode and 3 control biopsies from participants not experiencing rejection. Hypothesizing that IS type may influence rejection phenotype, we used ACR samples that included biopsies from 4 participants on tacrolimus, 3 on iscalimab, and 3 on belatacept maintenance IS (Table 1). Participants varied in terms of age, sex, race, etiology of end-stage renal disease, and donor type (living or deceased), and there were no significant differences in the number of HLA mismatches between IS groups (Supplemental Figure 1; supplemental material available online with this article; https://doi.org/10.1172/JCI170191DS1). Using our approach to collecting and freezing intact biopsies (29, 30) to allow for batch analyses and a cold-digestion protocol (31) to minimize temperature-driven artifacts in gene expression, biopsy-derived cells were subjected to scRNA-Seq. After alignment, quality control, and integration, uniform manifold approximation and projection (UMAP) analysis of all 13 index biopsies showed that cells were distributed across differing clusters with no clusters completely dominated by any particular sample (Figure 1A), indicating successful normalization and integration.

Initial cluster differentiation revealed 16 clusters of cells, including multiple immune and nonimmune kidney-derived cell populations. Cell types were identified based on differentially expressed genes (DEGs) and expression of canonical markers (32) (Supplemental Table 1). Within the immune cells, we observed γ/δ (clusters 0), CD4^+^ (cluster 1), and CD8^+^ (clusters 2–4), including a population of proliferating CD8^+^ and γ/δ (cluster 3) T cells; B cells (cluster 5); myeloid cells (clusters 6, 7); and plasmacytoid DCs (pDCs) (cluster 8) (Figure 1B). Of the kidney-derived cells, we identified proximal tubule (clusters 9–11), loop of Henle (cluster 12), distal tubule (clusters 13, 15), and endothelial (cluster 14) cells (Figure 1B). Individual gene expression plots across clusters were consistent with cell cluster definitions. Immune-cell clusters 0–8 expressed *PTPRC*, confirming that all immune clusters comprised leukocytes as well as other cell type–defining markers, including *TRDC* (clusters 0), *CD4* (cluster 1), *CD8A* (clusters 2–4), *KLRK1* (clusters 0–4), *ITGAX* (clusters 0, 7, 8), *CD19* (cluster 5), and *CD14* (clusters 7, 8) (Figure 1C). In humans, *CD4* is also expressed by myeloid cells (33), which we also observed (clusters 7, 8) (Figure 1C). As expected, no-rejection biopsies were dominated by kidney-derived cells, while rejection biopsies had prodigious and significantly increased amounts of immune infiltrates compared with the no-rejection samples (Figure 1, D and E), consistent with their rejection pathology score and histology (Supplemental Figure 2).

### CD8^+^ T cells dominate infiltrating immune-cell populations in rejecting kidney allografts.

**Figure 2:**
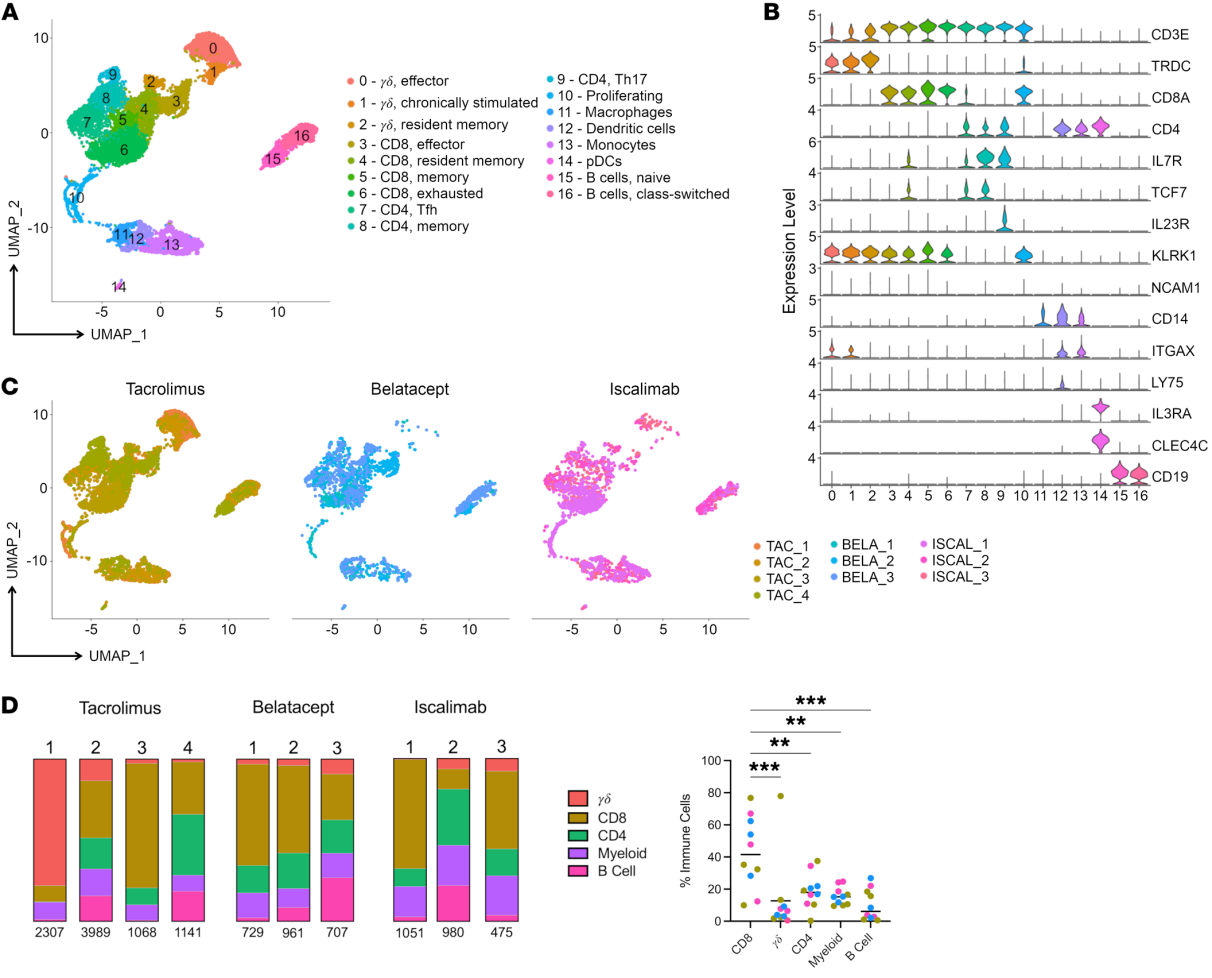
Diverse immune cells infiltrate during kidney allograft rejection. Index samples from the 10 participants undergoing rejection were integrated, clusters annotated as nonimmune cells were removed, and the data were renormalized and reclustered using Seurat. (**A**) UMAP plot shows immune-cell clusters and accompanying annotations. (**B**) Violin plots display the relative gene expression levels of indicated genes across each cluster. (**C**) Samples were segregated according to maintenance IS type. UMAP plots show immune-cell clustering of samples from participants with rejection under tacrolimus (left plot, shades of mustard), belatacept (middle plot, shades of blue), or iscalimab (right plot, shades of pink) maintenance IS. (**D**) Frequency of cell types within each sample displayed in bar graphs. Statistical analyses revealed a significantly increased proportion of CD8^+^ T cells in the immune infiltration as compared with other immune subtypes (*n* = 10). One-way ANOVA. ***P < 0.0006; **P < 0.007.

To further characterize immune infiltrates, immune-cell clusters from the 10 index rejection biopsies were subsetted and reanalyzed. Subsequent cellular annotations revealed the following: 3 γ/δ T cell clusters, including effector (cluster 0), chronically stimulated (cluster 1), and resident memory (cluster 2) populations; 4 CD8^+^ T cell clusters, including effector (cluster 3), resident memory (cluster 4), memory (cluster 5), and exhausted (cluster 6) populations; 3 CD4^+^ T cell clusters, including follicular helper (cluster 7), memory (cluster 8), and Th17 (cluster 9) cells; 4 myeloid clusters, including macrophages (cluster 11), DCs (cluster 12), extravascular monocytes (cluster 13), and pDCs (cluster 14); and 2 B cell populations, including naive (cluster 15) and class-switched (cluster 16) B cells (Figure 2, A and B, and Supplemental Table 2). Again, both CD8^+^ and γ/δ T cells were present in a proliferating T cell population (cluster 10).

To examine the influence of maintenance IS regimens on immune-cell types present within the allograft, cells were colored according to their IS regimens (tacrolimus [mustard], belatacept [blue], or iscalimab [pink]) (Figure 2C). Although one participant on tacrolimus IS had a dominant influx of γ/δ T cells, most immune-cell clusters were present at similar levels for all IS regimens (Figure 2D). Notably, all 10 samples were dominated by T cells, with significantly more CD8^+^ T cell infiltration compared with the rest of the immune-cell subtypes (Figure 2D). Overall, maintenance IS type did not grossly affect overall immune-cell composition of index biopsies.

### Intragraft CD8_EXP_ are heterogenous, including cells expressing different levels of markers associated with activation, exhaustion, and memory phenotypes.

**Figure 3:**
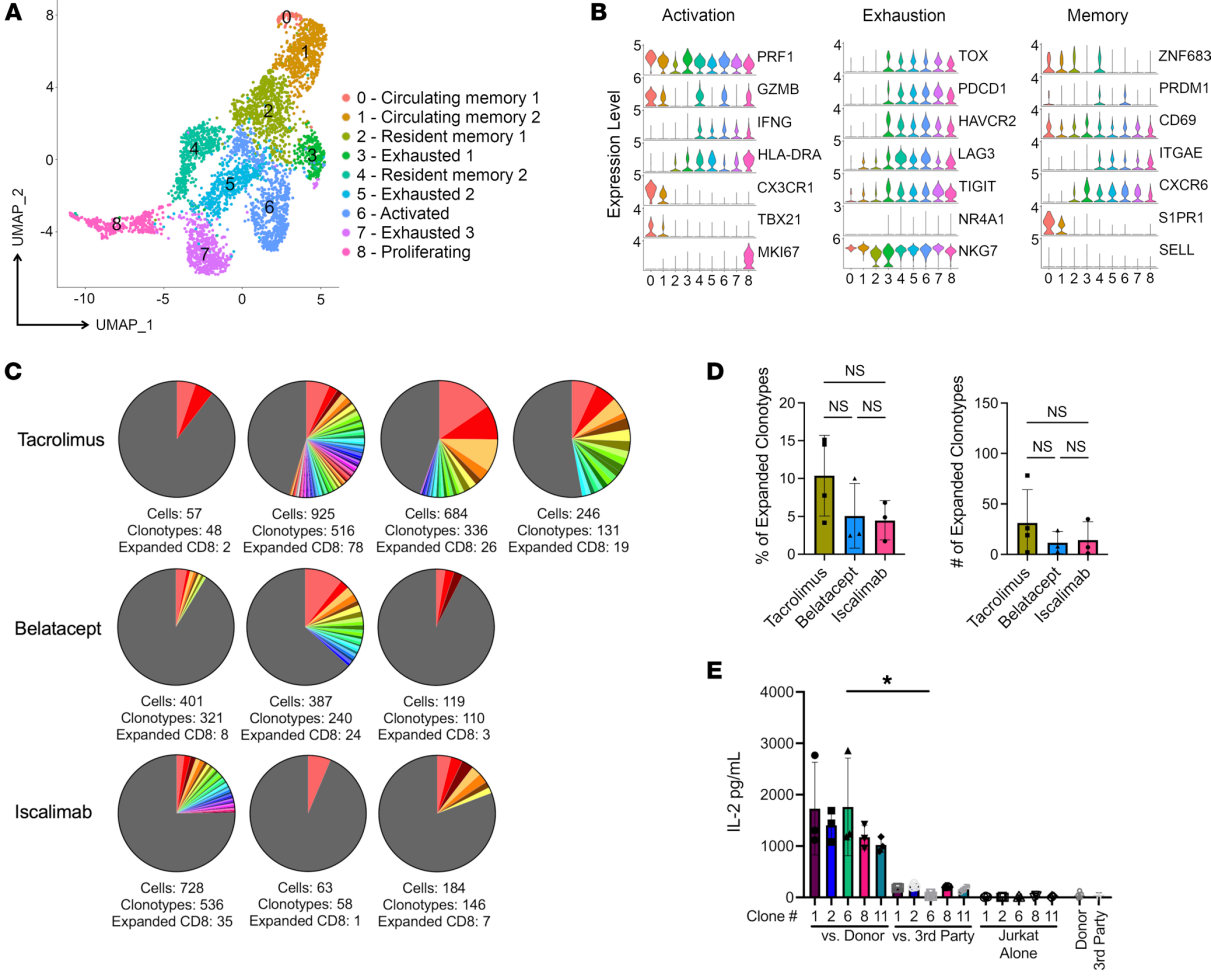
Analysis of infiltrating CD8^+^ T cells in kidney allograft rejection. CD8^+^ clusters from the immune-cell analysis were identified for further analyses; CD4^+^ and γ/δ T cells were removed. The samples were then reanalyzed using Seurat. (**A**) UMAP plot shows cell-type annotations based on DEGs. (**B**) Violin plots show relative expression levels of indicated genes selected to characterize cell-cluster phenotypes as activated, exhausted, and memory. (**C**) Pie charts display number and frequency of CD8_EXP_ found in the biopsy during rejection by participant sample, based on their unique CDR3α/β sequences. Expanded clonotypes are defined as having more than 2 cells with identical CDR3α/β sequences. Different colors represent individual expanded clonotypes (gray area represents unexpanded clonotypes), and the sizes of the colored areas represent the relative sizes of the expanded clonotypes. (**D**) Percentages (left graph) and total numbers (right graph) of CD8_EXP_ in each treatment group (tacrolimus, *n* = 4; belatacept, *n* = 3; iscalimab, *n* = 3) are displayed in the bar graphs (±SD). One-way ANOVA; NS, *P* > 0.05. (**E**) Full-length TCRs with unique CDR3α/β sequences derived from 5 CD8_EXP_ from 1 participant experiencing rejection (ISCAL_1) were subcloned into individual Jurkat 76 cells. Individual clones were cultured in triplicate either alone or with donor or third-party T cell–depleted PBMCs for 20 hours and IL-2 levels in the supernatant measured via ELISA. Results show the levels of IL-2 in pg/ml for each condition (±SD) done in triplicate (*n* = 3). One-way ANOVA. *P < 0.05.

As CD8^+^ T cells are primary drivers of ACR, analyses were refocused on just CD8^+^ T cells from index biopsies in the 10 participants with ACR (Figure 3A). To get greater clarity of the cellular phenotypes associated with each cluster, we compared their DEGs (Supplemental Table 3) as well as expression of markers associated with T cell activation and effector function (*PRF1*, *GZMB*, *IFNG*, *HLA-DRA*, *CX3CR1*, *TBX21*, and *MKI67*); exhaustion (*TOX*, *PDCD1*, *HAVCR2*, *LAG3*, *TIGIT*, *NR4A1*, and *NKG7*); and memory (*ZNF683*, *PRDM1*, *CD69*, *ITGAE*, *CXCR6*, *S1PR1*, and *SELL*) (Figure 3B). For example, in addition to their high-level expression of *PRF1* and *GZMB*, circulating memory CD8^+^ T cells (CD8_CIRCM_), which includes both effector and central memory cells (34), in clusters 1 and 2 expressed high levels of *S1PR1* (Figure 3, A and B), which promotes their tissue egress (34, 35). Cells in clusters 2 and 4 were defined as resident memory CD8^+^ T cells (CD8_RM_) based on their expression of *ZNF683*, *CD69*, and *CXCR6* (Figure 3, A and B), which are part of a tissue-residency genetic program (36). In the kidney, not all resident memory cells express *ITGAE* (37, 38). CD8_RM_ were further subdivided based on their differential expression of activation markers *GZMB*, *IFNG*, and *HLA-DRA* in cluster 4 relative to cluster 2 (Figure 3, A and B). Cells in clusters 3, 5, 6, and 7 were likely existing along a continuum of activation (CD8_ACTIV_) and exhaustion (CD8_EXH_) based on their expression of markers associated with exhaustion (39) (*TOX*, *HAVCR2*, *PDCD1*, *TIGIT*, *LAG3*) and varying expression of activation/effector function genes (HLA-DR, *GZMB*, and *IFNG)* (Figure 3, A and B). For example, cells in clusters 3, 5, and 7 may have been more exhausted, as they lacked expression of *GZMB* and had lower levels of *IFNG*, while cells in cluster 6 were more activated based on their higher expression of *GZMB* and *IFNG* (Figure 3, A and B). Finally, cluster 8 represents a population of proliferating CD8^+^ T cells (CD8_PROLIF_) based on expression of *MKI67* and other proliferation-associated genes (Figure 3, A and B, and Supplemental Table 3). Thus, allograft-infiltrating CD8^+^ T cells are heterogeneous, with phenotypes consistent with circulating memory, resident memory, and varying states of activation, exhaustion, and proliferation.

### Limited numbers of CD8_EXP_ are present in rejecting allografts.

CD8^+^ T cell clonality within the rejecting allograft was determined using the 10x Genomics Chromium Single Cell 5′ V(D)J platform. Full-length CDR3α/β sequences were obtained from approximately 90% of transcriptionally defined T cells, and expanded clonotypes were defined as a CDR3α/β paired sequence present on more than 2 cells. Strikingly, we found a limited number of CD8_EXP_ cells across all 3 IS modalities (average of 20 unique CD8_EXP_ per biopsy), while the majority of CD8^+^ T cells were unexpanded (CD8_UNEXP_) (Figure 3C). Further, there were no significant differences in the percentages or numbers of CD8_EXP_ between IS modalities (Figure 3D). Intriguingly, CD4^+^ clonal expansion was minimal, except for in participants TAC_4 and ISCAL_2, who had slightly higher proportions of CD4_EXP_ cells (Supplemental Figure 3). Surprisingly, the level of clonal expansion was not correlated with the number of HLA mismatches, rejection grade, or absolute lymphocyte count (ALC) (Supplemental Figure 4). While T cells can express 2 TCR-α chains, there was no significant difference in the percentage of T cells bearing 2 TCR-α chains between the expanded (7.1%) and unexpanded (8.9%) clonotypes, indicating clonal expansion is driven by antigen recognition by cells expressing a single TCR.

To further understand the donor specificities of CD8_EXP_, we arbitrarily chose 5 CD8_EXP_ CDR3α/β sequences from the scTCR-Seq data from one participant experiencing rejection (ISCAL_1) to subclone into Jurkat 76 cells (a thymoma cell line lacking endogenous TCR-α/β expression) (40). Resulting Jurkat 76 transfectants were cultured with T cell–depleted PBMCs from the recipient’s kidney donor or third-party cells and assayed for responses via IL-2 production. Strikingly, all 5 clones responded to donor, but not third-party, cells (Figure 3E) with significantly increased IL-2 production, confirming the alloreactivity of those CD8_EXP_ identified in the rejecting kidney biopsy.

### Maintenance IS type affects CD8_EXP_ gene expression.

**Figure 4:**
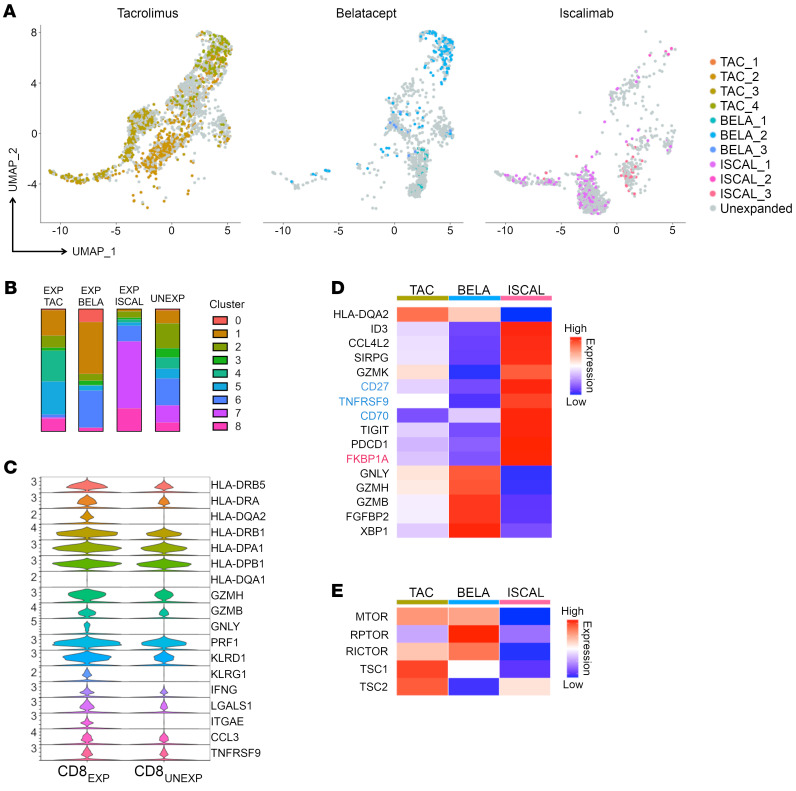
Gene expression differences in CD8_EXP_ among tacrolimus, belatacept, and iscalimab maintenance IS. (**A**) Clustering of CD8_EXP_ based on maintenance IS type. UMAP plots show clustering of CD8_EXP_ (colored dots) versus CD8_UNEXP_ (gray dots) from participants under either tacrolimus (left plot, shades of mustard); belatacept (right plot, shades of blue); or iscalimab (middle plot, shades of pink) maintenance IS. (**B**) Bar graphs display the fraction of expanded clonotypes (tacrolimus, belatacept, or iscalimab) and unexpanded clonotypes contributing to each CD8^+^ T cell cluster. (**C**) Violin plots show the relative expression of indicated genes in CD8_EXP_ and CD8_UNEXP_. (**D**) Heatmap displays (average) expression of unsupervised DEGs (*P* < 0.05) in CD8_EXP_ under tacrolimus (*n* = 4), belatacept (*n* = 3), and iscalimab (*n* = 3) maintenance IS. Blue text denotes 3 TNF family member genes, and red text denotes *FKBP1A*, a target of tacrolimus. (**E**) Heatmap displays a supervised analysis of the average expression of mTOR pathway–related genes in CD8_EXP_ from participants under tacrolimus, belatacept, and iscalimab maintenance IS.

Interestingly, cluster distribution of CD8_EXP_ varied based on maintenance IS. CD8_EXP_ from tacrolimus-treated participants were distributed across all clusters, but were more frequently represented in the CD8_RM_ and CD8_EXH_ populations (clusters 4, 5) than in CD8_EXP_ from belatacept- or iscalimab-treated participants (Figure 4, A and B). CD8_EXP_ from iscalimab-treated participants predominantly resided in another CD8_EXH_ population (cluster 7) and the CD8_PROLIF_ population (cluster 8), which was distinct from CD8_EXP_ from belatacept- or tacrolimus-treated participants (Figure 4, A and B). CD8_EXP_ from belatacept-treated participants clustered predominantly in both the CD8_CIRCM_ (cluster 1) and CD8_ACTIV_ (cluster 6) populations, consistent with prior work showing that BRR is associated with increased memory CD8^+^ T cells (5, 41). CD8_UNEXP_ were most represented in clusters with the lowest levels of CD8^+^ T cell activation (CD8_CIRCM_, CD8_RM_, and CD8_EXH_) (Figure 4, A and B). Further examination of the differential gene expression between CD8_EXP_ and CD8_UNEXP_ from all samples revealed that CD8_EXP_ had higher expression of *HLA* markers, activation, and effector markers (*GZMH*, *GZMB*, *GNLY*, *PRF1*, *KLRD1*, *KLRG1*, *IFNG*, and *ITGAE*), chemokines (*CCL3* and *CCL4*) and chemokine receptors (*CCL4L2*), and TNF family members *(TNFRSF9*) (Figure 4C). Thus, CD8_EXP_ express alloreactive TCRs and have gene expression consistent with cells that have undergone TCR-mediated activation.

We next examined the DEGs in CD8_EXP_ between the various IS modalities. CD8_EXP_ from participants treated with iscalimab had increased expression of TNF family members, such as *CD27*, *TNFRSF9*, and *CD70*, as well as *FKBP1A*, an intracellular tacrolimus-binding protein. Interestingly, *FKBP1A* expression is decreased in CD8_EXP_ under tacrolimus and belatacept IS (Figure 4D). In contrast, CD8_EXP_ from participants treated with belatacept showed upregulation of activation markers such as *GNLY*, *GZMH*, and *GZMB* when compared with iscalimab and tacrolimus CD8_EXP_ (Figure 4D).

Previously, our group demonstrated increased mTOR activity in peripheral blood CD8^+^ T cells in patients with ACR under belatacept, but not tacrolimus, and treatment of belatacept-refractory ACR with everolimus mitigated their ACR (5). Based on this, we performed a supervised analysis of mTOR pathway–related genes in the CD8_EXP_ under the 3 maintenance IS regimens (Figure 4E). Notably, CD8_EXP_ from participants under belatacept IS showed not only a significantly increased expression of mTOR complex genes (*mTOR*, *RPTOR*, and *RICTOR*), but also a decreased expression of 2 negative regulators of mTOR activation (*TSC1* and *TSC2*). This contrasts with CD8_EXP_ from participants under tacrolimus- or iscalimab-based IS, who showed lower levels of *RPTOR* and *RICTOR* and relatively higher levels of *TSC1* and *TSC2*. Combined, these data indicate that rejections arising under differing IS are associated with varying gene expression of potential therapeutic targets.

### CD8_EXP_ clonal populations may expand, contract, or persist in response to antirejection treatment.

**Figure 5:**
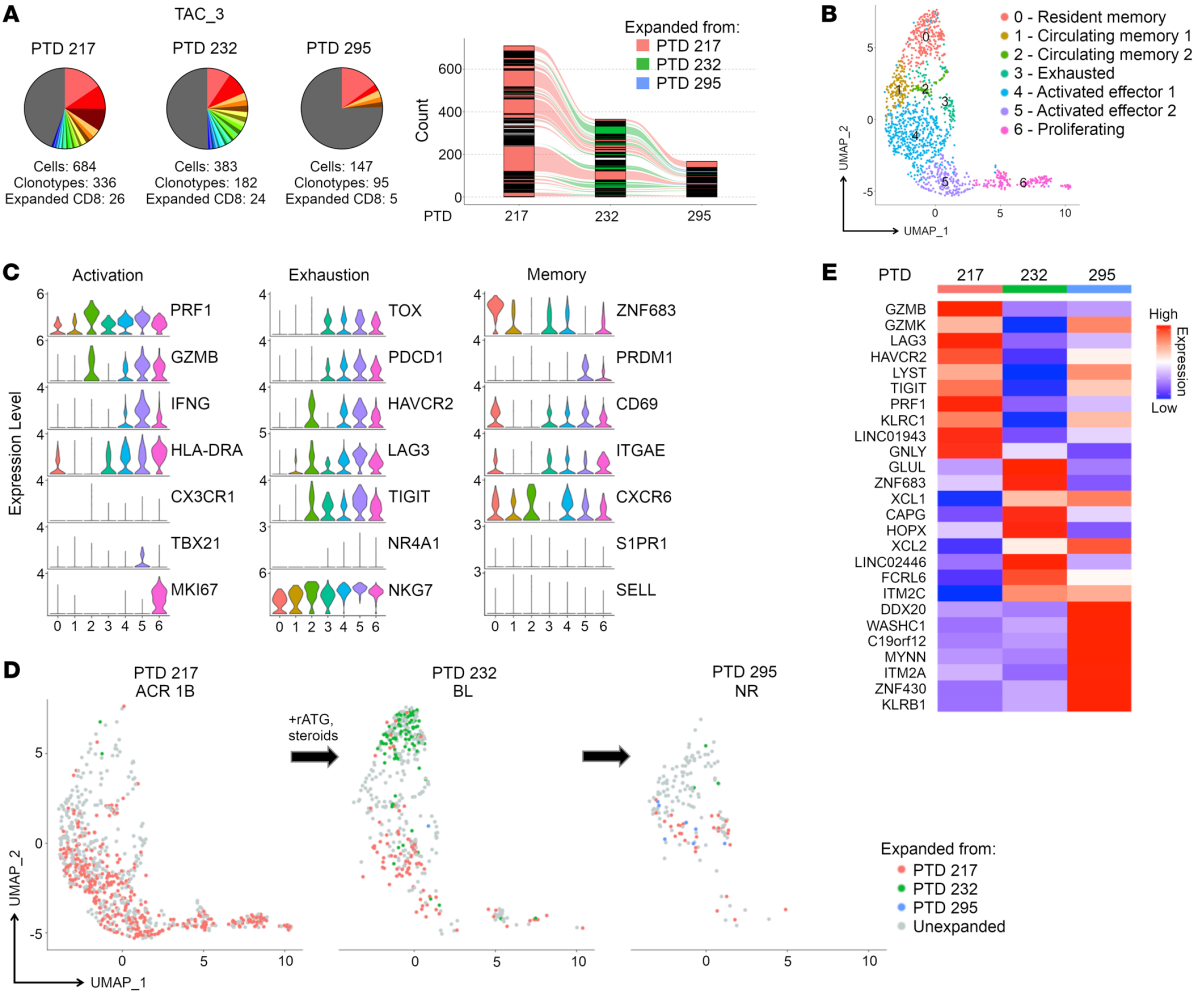
Temporal scRNA-Seq analysis of the response to antirejection therapy under tacrolimus maintenance IS. A participant on tacrolimus IS (TAC_3) was diagnosed with ACR 1B on PTD 217, and a biopsy was obtained prior to antirejection treatment with rATG and steroids. A second biopsy was obtained on PTD 232, and the participant was diagnosed with a borderline lesion. A third biopsy was taken at PTD 295, and the participant was diagnosed with no rejection. (**A**) Pie charts display number and frequency of expanded clonotypes found in the index biopsy (PTD 217) and subsequent follow-up biopsies (PTD 232, PTD 295). Bar graph shows overlapping clonotypes across the 3 time points. (**B**) UMAP shows CD8^+^ clusters in an integrated analysis of all time points. (**C**) Violin plots show relative expression levels of indicated genes selected to characterize cell cluster phenotypes as activated, exhausted, and memory. (**D**) Temporal analysis of CD8_EXP_ following antirejection therapy. UMAP plots show clustering of CD8_EXP_ (colored dots) versus CD8_UNEXP_ (gray dots) cells from the participant at PTD 217 (left plot), PTD 232 (middle plot), or PTD 295 (right plot). CD8_EXP_ first expanded on PTD 217 are shown in pink, those first expanding on PTD 232 are shown in green, and those first expanding on PTD 295 are shown in blue. (**E**) Heatmap shows average expression of unsupervised DEGs (*P* < 0.05) found between CD8_EXP_ at each time point.

We next examined the persistence of CD8_EXP_ clonal populations after antirejection therapy. Participant TAC_3 experienced an index Banff (42) ACR 1B rejection on posttransplant day (PTD) 217, which was treated with rabbit anti-thymocyte globulin (rATG) and prednisolone (Table 2). Two weeks later (PTD 232), a follow-up biopsy revealed histologic improvement to a Banff borderline lesion and a substantial decrease in the total numbers of CD8^+^ T cells. Intriguingly, scRNA-Seq analysis revealed an increase in the frequency of CD8_EXP_, from 7.7% (26 out of 336 total clonotypes) in the index biopsy to 13.2% (24 out of 182) (Figure 5A). Out of the 24 CD8_EXP_ that were identified at PTD 232, 10 were identical to those present as CD8_EXP_ in the index biopsy (PTD 217) (Figure 5A). An integrated analysis of all 3 time points revealed 7 CD8^+^ T cell clusters, including 1 CD8_RM_ (cluster 0), CD8_CIRCM_ (clusters 1, 2), CD8_EXH_ (cluster 3), 2 CD8_ACTIV_ (clusters 4, 5), and CD8_PROLIF_ (cluster 6) (Figure 5, B and C, and Supplemental Table 4). On the index biopsy, most CD8_EXP_ were CD8_ACTIV_ (clusters 4, 5) and CD8_PROLIF_ (cluster 6). Rejection treatment with rATG and corticosteroids resulted in marked reduction in CD8_ACTIV_ (clusters 4, 5) and CD8_PROLIF_ (cluster 6) (Figure 5D and Supplemental Table 4). Notably, at PTD 232, a dominant CD8_EXP_ population with a CD8_RM_ phenotype (cluster 0) appeared. Nine weeks later (PTD 295), another biopsy revealed no histologic rejection and a loss of the CD8_RM_ phenotype (cluster 0) as well as further reduction in CD8_ACTIV_ (clusters 4, 5) and CD8_PROLIF_ (cluster 6), but a small increase in CD8_EXH_ (cluster 3). scTCR-Seq analysis revealed 5 distinct CD8_EXP_, 3 of which were from previous biopsies (Figure 5, A and D). DEG analysis between the CD8_EXP_ from the 3 time points revealed that CD8_EXP_ from the index rejection biopsy (PTD 217) expressed effector function genes (*GZMB*, *GZMK*, and *GNLY*), but also exhaustion markers (*LAG3*, *HAVCR2*, and *TIGIT*), while clonotypes 2 weeks later (PTD 232) displayed resident memory genes (*ZNF683*, *CD69*, and *ITGAE)* and clonotypes 2 months later (PTD 295) expressed *GZMK* and *KLRB1* as well as chemokines *XCL1* and *XCL2* (Figure 5E). Thus, even though histologic rejection resolved, CD8_EXP_ persisted at 11 weeks after initial rejection despite rATG and corticosteroid antirejection treatment.

**Figure 6:**
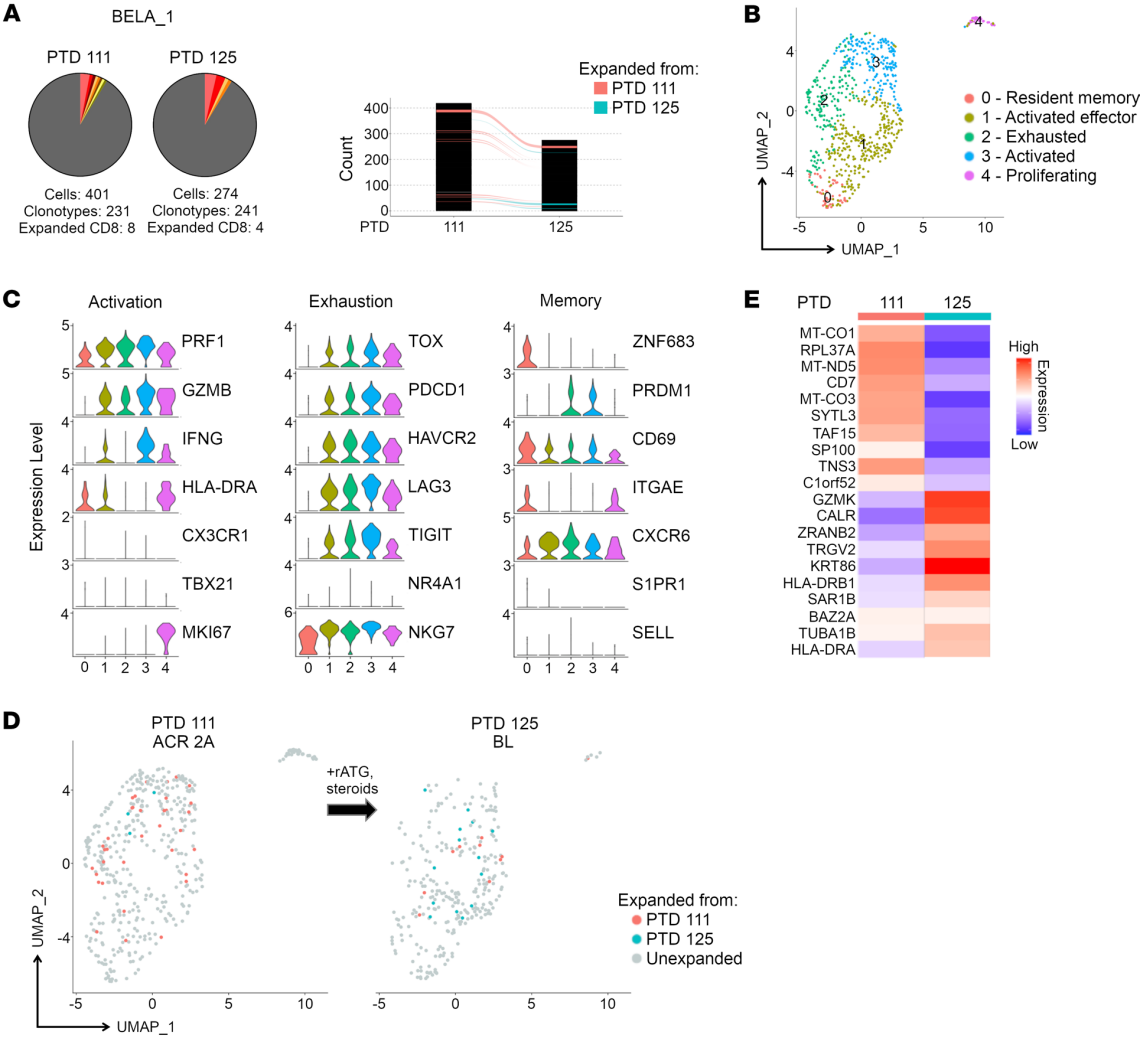
Temporal scRNA-Seq analysis of the response to antirejection therapy under belatacept maintenance IS. A participant on belatacept IS (BELA_1) was diagnosed with ACR 2A on PTD 111 and a biopsy was obtained prior to antirejection treatment with rATG and steroids. A second biopsy was obtained on PTD 125, and the participant was diagnosed with a borderline lesion. (**A**) Pie charts display number and frequency of expanded clonotypes found in the index biopsy (PTD 111) and the subsequent follow-up biopsy (PTD 125). Bar graph shows overlapping clonotypes across the 2 time points. (**B**) UMAP shows CD8^+^ clusters in an integrated analysis of both time points. (**C**) Violin plots show relative expression levels of indicated genes selected to characterize cell cluster phenotypes as activated, exhausted, and memory. (**D**) Temporal analysis of CD8_EXP_ following antirejection therapy. UMAP plots show clustering of CD8_EXP_ (colored dots) versus CD8_UNEXP_ (gray dots) from the participant at PTD 111 (left plot) and PTD 125 (right plot). CD8_EXP_ first expanded on PTD 111 are shown in pink, and those first expanding on PTD 125 are shown in blue. (**E**) Heatmap shows average expression of unsupervised DEGs found between CD8_EXP_ at each time point.

We also followed participant BELA_1, for whom a biopsy on PTD 111 revealed a Banff ACR 2A rejection under belatacept-based IS. This rejection episode was treated with rATG and corticosteroids (Table 2), and 2 weeks later (PTD 125), a follow-up biopsy revealed improvement to a borderline lesion. Interestingly, despite histological improvement and a decrease in the number of CD8_EXP_ from PTD 111 (8 CD8_EXP_) to PTD 125 (4 CD8_EXP_), the frequency of total clonotypes and number of expanded cells were similar between the 2 time points (Figure 6A). Of the 4 CD8_EXP_ identified at PTD 125, 1 was previously identified from PTD 111 while 3 were newly expanded at PTD 125 (Figure 6, A and D). An integrated analysis of CD8^+^ T cells from both time points revealed 5 distinct clusters, including CD8_RM_ (cluster 0), CD8_ACTIV_ (clusters 1, 3), CD8_EXH_ (cluster 2), and CD8_PROLIF_ (cluster 4) (Figure 6, B and C, and Supplemental Table 5). In-depth analysis of the gene expression of CD8_EXP_ following antirejection treatment with rATG and corticosteroids revealed surprisingly limited changes in DEGs despite histologic improvement to a borderline lesion (Figure 6E).

**Figure 7:**
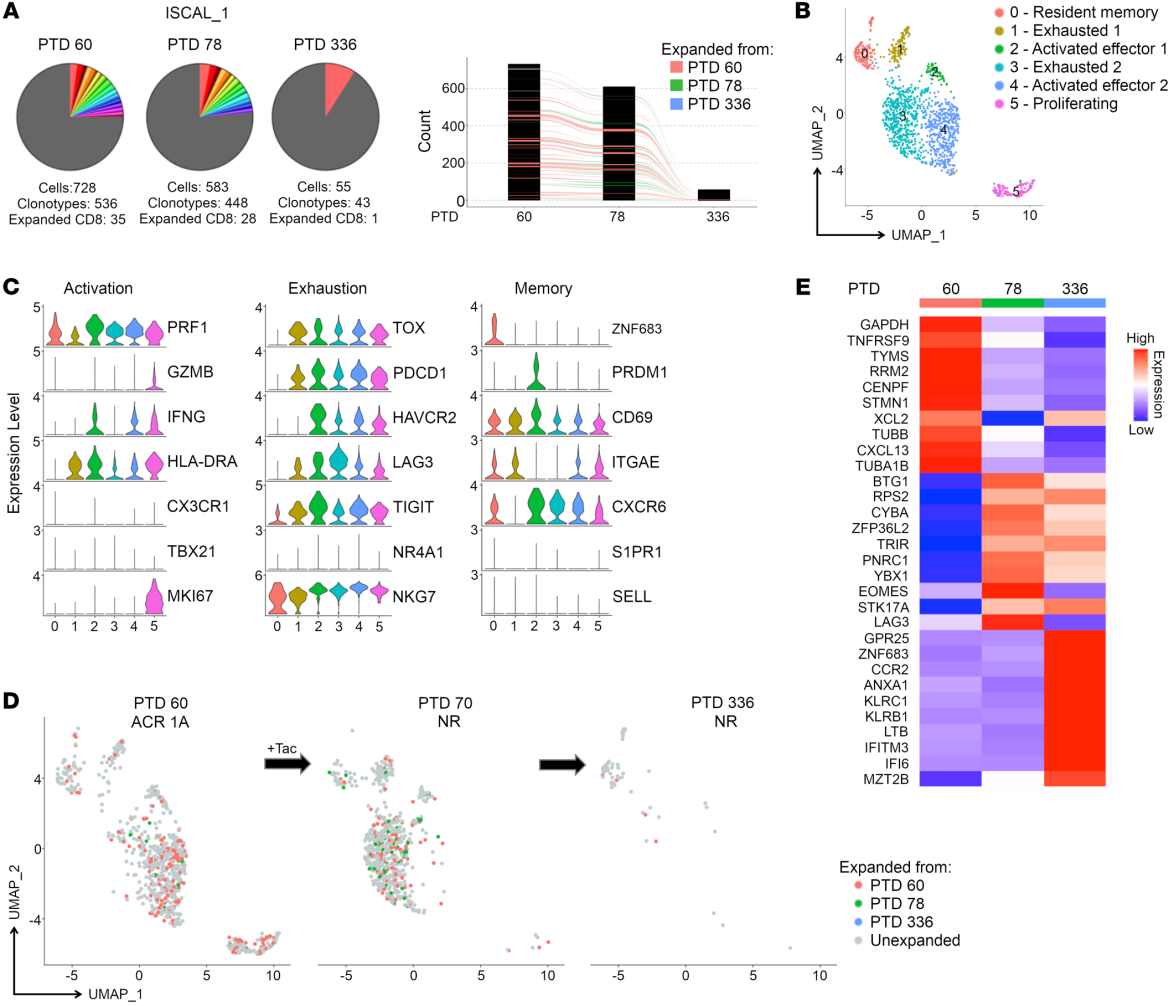
Temporal scRNA-Seq analysis of the response to antirejection therapy under iscalimab maintenance IS. A participant on iscalimab IS (ISCAL_1) was diagnosed with ACR 1A on PTD 60, and a biopsy was obtained prior to antirejection treatment with tacrolimus. A second biopsy was obtained on PTD 78, and the participant was diagnosed with no rejection. A third biopsy was taken at PTD 336, and the participant was again diagnosed with no rejection. (**A**) Pie charts display number and frequency of expanded clonotypes found in the index biopsy (PTD 60) and subsequent follow-up biopsies (PTD 78, PTD 336). Bar graph shows overlapping clonotypes across the 3 time points. (**B**) UMAP shows CD8^+^ clusters in an integrated analysis of all time points. (**C**) Violin plots show relative expression levels of indicated genes selected to characterize cell cluster phenotypes as activated, exhausted, and memory. (**D**) Temporal analysis of CD8_EXP_ following antirejection therapy. UMAP plots show clustering of CD8_EXP_ (colored dots) versus CD8_UNEXP_ (gray dots) from the participant at PTD 60 (left plot), PTD 78 (middle plot), or PTD 336 (right plot). CD8_EXP_ emerging on PTD 60 are shown in pink, those emerging on PTD 78 are shown in green, and those emerging on PTD 336 are shown in blue. (**E**) Heatmap shows average expression of unsupervised DEGs (*P* < 0.05) found between CD8_EXP_ at each time point.

Participant ISCAL_1 presented with a Banff ACR 2A rejection under iscalimab-based IS at 1 month after transplant that was treated with rATG and a corticosteroid taper for persisting Banff 1B rejection. A biopsy obtained on PTD 60 revealed a Banff 1A rejection (Table 2), and scTCR-Seq analysis revealed 35 individual CD8_EXP_. Tacrolimus-based antirejection treatment was initiated, and a biopsy obtained 2.5 weeks later (PTD 78) demonstrated no rejection and significantly reduced CD8_EXP_. However, there was no change in the frequency of CD8_EXP_ (6.5% at the first time point and 6.3% at follow-up), and nearly half of the CD8_EXP_ were identical to those in the first biopsy (Figure 7, A and D). Following rejection resolution, tacrolimus was tapered. A repeat biopsy obtained approximately 1 year later (PTD 336) revealed histologic resolution of rejection, and only 1 CD8_EXP_ persisted from the initial time point (PTD 60) (Figure 7, A and D). An integrated analysis of CD8^+^ T cells from all 3 time points identified 6 clusters: CD8_RM_ (cluster 0), CD8_EXH_ (clusters 1, 3), CD8_ACTIV_ (clusters 2, 4), and CD8_PROLIF_ (cluster 5). Most of the CD8_EXP_ identified from PTD 60 were present as CD8_ACTIV_ (cluster 4) and CD8_PROLIF_ (cluster 5) with a few as CD8_RM_ (cluster 0) and a few as CD8_EXH_ (cluster 1) (Figure 7, B and C, and Supplemental Table 6). Strikingly, at PTD 78, nearly all CD8_EXP_ that were CD8_ACTIV_ (cluster 4) were decreased and a substantial number of those clones were now present as CD8_EXH_ (cluster 3) (Figure 7, B and C, and Supplemental Table 6). Notably, despite rejection resolution at PTD 336, the one remaining CD8_EXP_ first identified from rejection at PTD 60 had a CD8_RM_ phenotype (evidenced by *ZNF683* expression) (cluster 0) (Figure 7E). Notably, the TCR expressed by this clone was one of the TCRs defined as alloreactive (Figure 3D). This demonstrates that, despite resolution of rejection, alloreactive CD8^+^ T cell clones can persist for at least a year within the histologically normal allograft.

**Figure 8:**
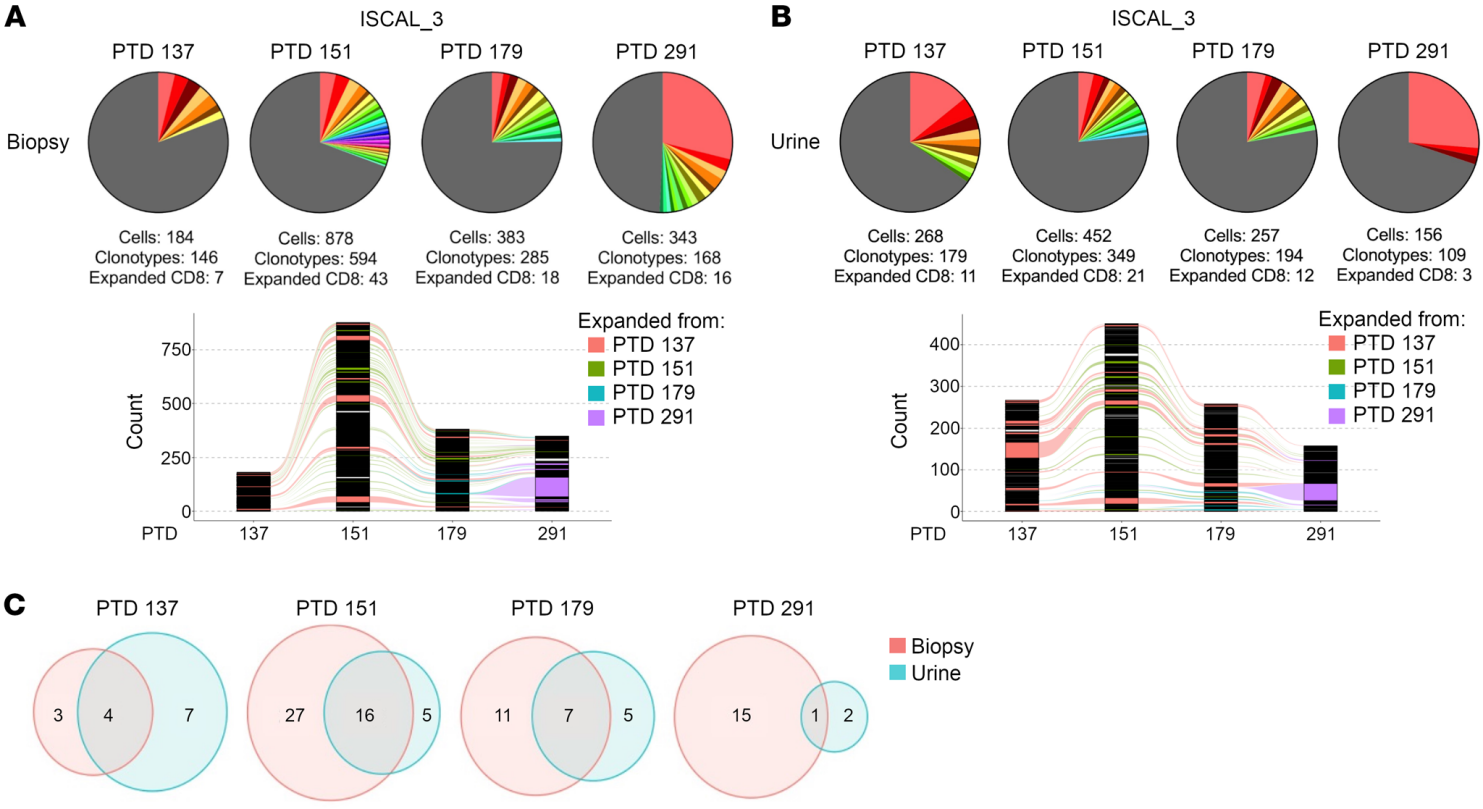
Comparison of CD8_EXP_ between the biopsy and paired urine samples in a participant undergoing treatment-refractory rejection. A participant on iscalimab IS (ISCAL_3) was diagnosed with ACR 1B on PTD 137, and a biopsy was obtained prior to antirejection treatment with tacrolimus conversion and steroids. A second biopsy was obtained on PTD 151, and the participant was diagnosed with ACR 1B. MMF was then added to the antirejection regimen. A third biopsy was taken at PTD 179, and the participant was diagnosed as borderline and MMF was tapered off. A final biopsy was taken at PTD 291 and showed mixed 1B rejection. (**A** and **B**) Pie charts (top) display number and frequency of expanded clonotypes found at each biopsy (**A**) and urine (**B**) sample, and bar graphs (bottom) display clonotypes found at the indicated time points. Different colors represent individual expanded clonotypes (gray area represents unexpanded clonotypes), and the size of the colored area represents the relative size of the expanded clonotypes. (**C**) Venn diagrams display overlap of individual CD8_EXP_ clonotypes between biopsies and their paired urine sample at the indicated time points.

A fourth participant, ISCAL_3, was diagnosed with a Banff 1B rejection at 20 weeks after transplant (PTD 137) (Table 2) while on iscalimab maintenance IS, and scTCR-Seq analysis revealed 7 distinct CD8_EXP_ (Figure 8A). Interestingly, scTCR-Seq analysis of urine sediment at the same time point contained 11 CD8_EXP_, 4 of which were identical to the CD8_EXP_ in the biopsy (Figure 8, B and C). To treat their rejection, the participant was converted to tacrolimus IS, given a prednisolone pulse, and iscalimab was discontinued. Two weeks later (PTD 151), a repeat biopsy revealed Banff ACR 1B rejection (Table 2) and an increase in CD8_EXP_ clonal frequency (4.7% to 7.2%). Importantly, each CD8_EXP_ present in the index biopsy was also observed in the second biopsy, and 16 CD8_EXP_ were found in both the biopsy and urine samples (Figure 8, A–C). Four weeks later (PTD 179), repeat biopsy revealed a borderline lesion (Table 2), with a continued persistence of CD8_EXP_ (6.3%) (Figure 8A). Approximately 72% (13 out of 18) of CD8_EXP_ in the PTD 179 biopsy had been observed in prior biopsies and a similar persistence of CD8_EXP_ was observed in the urine (Figure 8, A and B). An approximate 50% overlap in CD8_EXP_ was noted in both biopsy and urine at PTD 179 (Figure 8C). Four months later (PTD 291), the participant was diagnosed with a Banff 1B mixed acute rejection. scTCR-Seq again demonstrated persistence of CD8_EXP_ at a frequency of 9.5% of all clonotypes (16 out of 168), and roughly half of these were observed in earlier biopsies (Figure 8C). Overall, these data show that in addition to persistent CD8_EXP_ in the rejecting allograft despite antirejection therapy, persistent CD8_EXP_ can also be observed in the urine, and urine CD8_EXP_ reflect those found in the graft.

**Figure 9:**
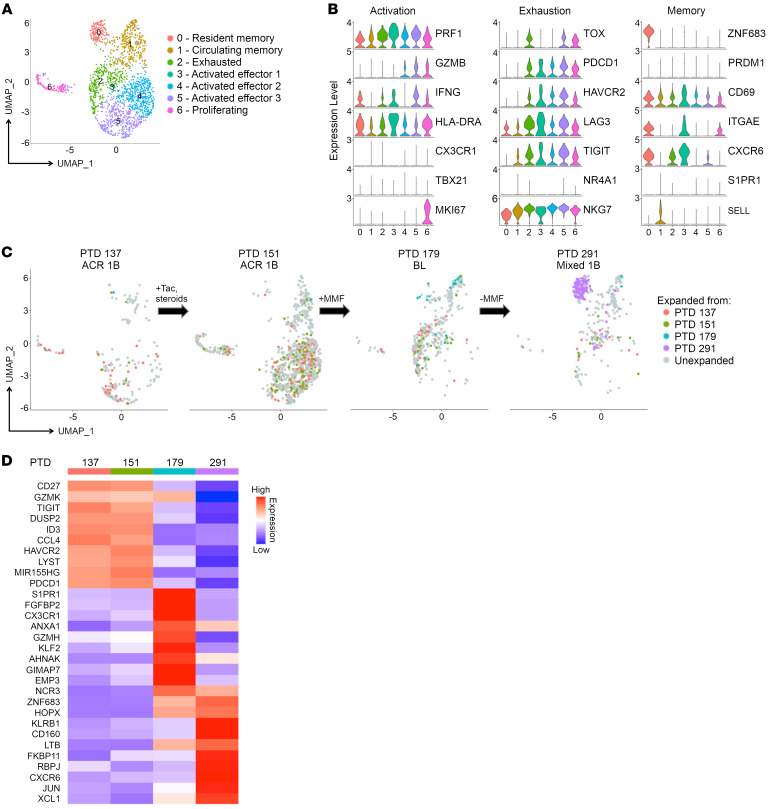
Temporal scRNA-Seq analysis of treatment-refractory rejection under iscalimab maintenance IS. (**A**) Allograft-derived CD8^+^ T cells from all time points from participant ISCAL_3 were integrated and renormalized, and UMAP plot shows individual CD8^+^ clusters based on DEGs. Note that some clusters are unique to individual time points. (**B**) Violin plots show relative expression levels of indicated genes selected to characterize cell cluster phenotypes as activated, exhausted, and memory. (**C**) Temporal analysis of CD8_EXP_ during treatment-refractory rejection therapy. UMAP plots show clustering of CD8_EXP_ (colored dots) versus CD8_UNEXP_ (gray dots) from the participant at PTD 137 (left plot), PTD 151 (middle left plot), PTD 179 (middle right plot), or PTD 291 (right plot). CD8_EXP_ emerging on PTD 137 are shown in pink, on PTD 151 are shown in green, on PTD 179 are shown in blue, and those emerging on PTD 291 are shown in purple. (**D**) Heatmap shows average expression of unsupervised DEGs found between expanded clonotypes at each time point.

An integrated analysis of allograft-resident CD8^+^ T cells from all 4 time points revealed 7 clusters, including CD8_RM_ (cluster 0), CD8_CIRC_ (cluster 1), CD8_EXH_ (cluster 2), CD8_ACTIV_ (clusters 3–5), and CD8_PROLIF_ (cluster 6) (Figure 9, A and B, and Supplemental Table 7). Most CD8_EXP_ from the index biopsy (PTD 137) had gene expression consistent with CD8_ACTIV_ (clusters 4, 5) and CD8_PROLIF_ (cluster 6) (Figure 9, A–C), and after addition of tacrolimus and corticosteroids for antirejection therapy, most CD8_EXP_ from the second biopsy (PTD 151) were identified as CD8_CIRCM_ (cluster 1) and CD8_ACTIV/EFF_ (clusters 4, 5) (Figure 9, A–C). Mycophenolate mofetil (MMF) was added to the maintenance IS regimen and another biopsy taken at PTD 179. The majority of CD8_EXP_ at this time point appeared to have shifted their gene expression and were identified as CD8_EXH_ cells (cluster 2), with some cells having a CD8_CIRCM_ (cluster 1) and a CD8_RM_ (cluster 0) phenotype (Figure 9, A–C). Finally, at PTD 291 following tapering of MMF, a new clonotype appeared and localized to cluster 0, a cluster populated by only a few cells in the earlier samples and whose gene expression profile was consistent with a pathogenic CD8_RM_ phenotype (cluster 0), showing high levels of *ZNF683* and *CD160* (Figure 9, C and D). Taken together, these data show that, during unresolved rejection, treatment with tacrolimus, corticosteroids, and MMF failed to eliminate CD8_EXP_ and instead was associated with substantial changes in their gene expression.

## Discussion

Using single-cell genomics, we uncovered an unexpectedly small number of CD8_EXP_ present in rBx undergoing rejection. This seemingly contrasts with prior data using bulk TCR-β sequencing approaches of kidney allograft biopsies (16, 17, 20, 43). We envision a few explanations for these differences. First, transplant patients are often lymphopenic due to induction therapy, which could severely limit the available repertoire. Although we found that patients with a normal ALC had low numbers of CD8_EXP_, it remains possible that induction therapy drives a long-lasting reduction of the available repertoire. Second, while it is possible that many CD8_UNEXP_ are alloreactive, we think this unlikely, as our longitudinal analysis showed that very few CD8_UNEXP_ ended up as CD8_EXP_ in subsequent biopsies. Third, alloresponses may involve immunodominance mechanisms. For example, perhaps the nature of the T cell (i.e., preexisting memory or TCR avidity) allows a limited number of clonotypes to outcompete for resources and dominate the response. Further work determining TCR specificity of CD8_EXP_ and their presence in naive and memory T cell populations prior to transplantation will help distinguish between these possibilities.

In this regard, our platform of subcloning of TCRs into Jurkat 76 cells and testing them against donor versus third-party T cell–depleted PBMCs will be useful for studying the biology of allospecific TCRs. In addition to linking TCR specificity to cell phenotype, screening combinatorial peptide libraries (CPLs) will enable the identification of peptides bound to donor HLA recognized by allospecific TCRs. This is important because a lack of an ability to track and monitor allospecific T cells has been a major impediment to understanding their development and function. Further, as many have suggested, a large fraction of allospecific T cells may be preexisting memory cells with specificities to pathogens previously encountered (44, 45). Similarly, use of *recipient* PBMCs and CPLs will allow for identification of TCRs with potential cross reactivity. Because of this, future extensions of these studies have the potential to yield insights into the fundamental nature of allorecognition, whether alloreactive TCRs focus on HLA epitopes versus peptide, and protein structure/function analysis of TCR CDR3/HLA/peptide interactions. In addition, further work will be necessary to determine the function of graft-resident T cells and further connect transcriptomic phenotype with function and potentially with specificity.

Our data also showed that the type of IS affected the gene expression and phenotype of CD8_EXP_. Importantly, scRNA-Seq provided the resolution to identify potential therapeutic targets that may be exploited for optimal antirejection therapy. For example, we previously reported that patients undergoing BRR responded to treatment with everolimus (5). Our data here are consistent with an underlying mechanism for this prior observation, as CD8_EXP_undergoing BRR have elevated expression of mTOR components *RICTOR* and *RPTOR*. Similarly, increased expression of *FKBP1A* in iscalimab-refractory rejection suggests that such rejections may be sensitive to tacrolimus. Thus, such analysis could provide physicians with informed and personalized approaches to optimize antirejection therapies.

We also found that CD8_EXP_ can persist in the kidney allograft for months, despite successful antirejection therapy, confirming and extending prior mixed lymphocyte reaction (MLR) studies in peripheral blood (16). Interestingly, we also found that individual CD8_EXP_ surviving rejection therapy adapt, and possibly survive, by altering gene expression (i.e., adopting a CD8_RM_ phenotype) in response to antirejection therapy. This incomplete CD8_EXP_ elimination and persistence may underlie recurrent rejection and/or long-term smoldering allograft injury, contributing to rejection-associated reduction in allograft survival. Additional work is required to determine the persistence and specificity of TCRs of CD8_EXP_ from participants undergoing antirejection therapy and whether these CD8_RM_ cells are associated with protective (46) versus pathologic (47) responses. An intriguing question remains — whether complete elimination of CD8_EXP_ will substantially improve the poor allograft survival rates that are observed following treatment of moderate and severe ACR. Addressing this issue may represent a major advance in rejection therapy.

Our observation that clonally identical CD8_EXP_ are found in the urine and biopsy is likely a result of CD8^+^ T cell killing of renal tubular epithelial cells, underlying the predominant histologic feature of ACR (tubulitis). In tubulitis lesions, CD8_EXP_ likely traverse the renal tubular basement membrane and gain access to the urinary space, in contrast with those remaining in the interstitial space. We are currently determining the number and gene expression of CD8_EXP_ in the urine and whether this correlates with the degree of tubulitis. Interestingly, for participant ISCAL_3, the CD8_EXP_ with a CD8_RM_ phenotype was present only in the biopsy (not urine), suggesting their retention in the kidney precluded their ability to traverse the basement membrane.

While we focused on CD8^+^ T cells, many other cell types are amenable to similar analysis. In this light, there was one participant (TAC_1) with a substantial influx of γδ T cells, and although it is possible that this massive influx of γδT cells could be due to CMV viremia (48, 49), this patient was CMV^+^ but was never demonstrated to have CMV viremia. Further, we note that despite an exhaustive search, we have not been able to confidently identify a population of NK cells in our samples, as we cannot be confident any identified population are not αβ or γδ T cells that were dropouts for TCR expression. Therefore, in-depth analyses of other immune-cell and kidney-derived cell populations are currently underway utilizing the same samples described herein. In addition, other single-cell genomics-based approaches, including spatial transcriptomics (ST) and assay for transposase-accessible chromatin with sequencing (ATAC-Seq) ST will place the cell populations identified by scRNA-Seq in their histologic context, while ATAC-Seq may further refine and expand our knowledge of gene regulatory networks active in various cell types.

In summary, scRNA-Seq analysis of human renal allograft rejection reveals highly restricted CD8_EXP_ that exhibit alloreactivity and varying responses to rejection therapy, including their persistence despite rejection resolution. Importantly, these CD8_EXP_ vary in gene expression based on the nature of maintenance IS. These fundamental insights delineate approaches for developing innovative individualized rejection therapies that can be tested in carefully designed clinical trials.

## Methods

### Sample collection.

Tissue samples were collected directly during the biopsy procedure using an 18-gauge biopsy needle and placed immediately in HypoThermosol FRS Preservation Solution (HTS) (BioLife Solutions Inc., 101102) on ice. When applicable, clean-catch urine samples were collected and spun down at 300*g* to obtain the cellular components. Both sample types were frozen at –80°C in CryoStor CS10 (BioLife Solutions Inc., 210102) in a Mr. Frosty Freezing Container (Nalgene, 5100-0001), then stored in liquid nitrogen until ready for analysis.

### Tissue dissociation.

Tissue dissociation protocol was modified from a previously described cold-active protease digestion (31). Kidney core biopsies were slowly thawed, cut into 1 to 2 mm pieces, then subjected to gentle cold digestion on ice using 10% FBS-supplemented RPMI media containing 100 mg/mL trypsin inhibitor from soybean (Roche, 10109886001), 10 mg/mL collagenase A from clostridium histolyticum (Roche, 10103586001), 10 mg/mL collagenase type IV from clostridium histolyticum (Worthington, LS004186), 250U DNase I (Roche, 4536282001), and 5 mM CaCl_2_. Digestion was performed twice at 10 minutes each, with intermittent rotating and gentle pipette mixing with wide orifice tips. The digested tissue was passed through a preprimed 30 mm cell strainer, breaking up any remaining visible tissue using a rubber syringe plunger. The single-cell suspension was passed through a second preprimed 30 mm cell strainer, then centrifuged at 300*g* at 4°C for 5 minutes. Viability and live cells were counted by Trypan blue exclusion, then resuspended at 1,000 cells/mL per the 10x Genomics Chromium protocol. The cells, kept on ice, were immediately prepared for single-cell barcoding.

### Single-cell barcoding, cDNA synthesis, and library preparation.

All samples were processed for single-cell sequencing following the Chromium Next GEM Single Cell V(D)J Reagent Kit, version 1.1, protocol. Briefly, cells were uniquely barcoded by using 10x fluidics (10x Genomics Chromium Single Cell Controller) to combine each individual cell with an individual barcoded Single Cell 5′ Gel Bead creating a Gel Beads-in-Emulsion (GEMs) solution (10x Genomics, PN-1000165 and PN-1000120). An average of 17,400 cells were loaded to achieve an estimated 10,000 cell recovery. GEM gel beads were dissolved, and cDNA was synthesized from the resulting tagged mRNA transcripts over 14 amplification cycles; 50 ng of cDNA was used for the construction of each library. Total gene expression libraries (PN-1000020) and libraries of enriched TCR sequences (PN-1000005) were created using the Single Index Kit Set A (PN-1000213).

### Sequencing, alignment, and generation of matrices.

Total gene expression and TCR sequence–enriched libraries were sequenced on the NovaSeq 6000 sequencer using S1, S2, or S4 flow cells, with the goal of obtaining more than 320M reads per sample. Raw base call files were demultiplexed with Cell Ranger (version 6.1.2) using mkfastq. Reads were aligned to human reference genome (version GRCh38) and gene expression quantified against GENCODE (release 32) (https://www.gencodegenes.org/) using the count function of CellRanger.

### scRNA-Seq analysis pipeline.

Single-cell analysis was carried out with R (version 4.2.0) running inside RStudio (version 4.1.1) using Seurat (version 4.1.0) (50, 51). Cells expressing more than 25% mitochondrial gene transcripts or fewer than 200 genes, including additional low-quality cells, were excluded from the analysis. TCR-α and TCR-β gene variants were collapsed as singular TRA and TRB genes, respectively. Gene expression counts were normalized with the NormalizeData function in Seurat. The samples were integrated using FindIntegrationAnchors and IntegrateData functions from Seurat. This integrated data set was used for principal component analysis, variable gene identification, Shared Nearest Neighbor (SNN) clustering analysis, and creation of UMAP. Metadata were updated to include identities of TCR clonotypes and those categorized as expanded and unexpanded, as described below. DEGs were determined using FindMarkers, with a logfc threshold of 1 and minimum percentage expression of 0.2. Genes that were differentially expressed at an adjusted *P* value of less than 0.05 were used for analyses.

### TCR clonal analysis.

Cell Ranger outputs for the TCR-α/β sequencing data were merged into the Seurat metadata for various integrated analyses. Filtered_contig_annotations.csv and clonotypes.csv files were used to obtain CDR3α/β information linked to individual barcodes, which were then merged with barcodes from the Seurat metadata to combine scRNA-Seq analysis with scTCR-Seq analysis. CD8^+^ cells were identified from the immune-cell populations through subsetting of CD8^+^ clusters and removal of cells expressing *CD4*, *TRDC*, and *CD68*. Clonotypes with identical CDR3α/β sequences present in more than 2 cells (identified through unique barcodes) were determined to be expanded. Clonotypes with 2 CDR3β chains or only an individual CDR3α or CDR3β chain were classified as unexpanded. Analysis of the merged Seurat metadata allowed determination of numbers of CD8^+^ barcodes, total numbers of clonotypes, and numbers of CD8_EXP_ and CD8_UNEXP_ clonotypes.

To determine the position of CD8_EXP_ clonotypes on UMAPs, expanded clonotypes were recalled using their clonotype_id and set as a new identity on the plots. Overlapping clonotypes between biopsy and urine samples were done using the package VennDiagram, and clonotype tracking over sequential time points was done using the package Immunarch (52), version 0.9.0, with modified input files to reflect only CD8^+^ cells in the analysis.

### TCR-expressing Jurkats.

TCR-α/β^–/–^ Jurkat 76 cell lines were provided by Michael Nishimura (Loyola University, Chicago, Illinois, USA). Jurkat 76 cells stably expressing the TCRs of interest were generated by transfection using the Neon Transfection System (Thermo Fisher). The cells were transfected with the pCMV(CAT)T7-SB100 plasmid and the pSBbi-Neo Sleeping Beauty vector containing the full-length TCR-α and -β chains (obtained from scTCR-Seq of 1 participant [ISCAL_1] undergoing rejection) separated by the P2A self-cleaving peptide as previously described (53). After electroporation, the cells were maintained in RPMI media (RPMI 1640 with 10% FBS, 100 units/mL penicillin, and 100 μg/mL streptomycin). and were incubated at 37°C and 5% CO_2_. Cells expressing the TCR of interest were selected for by using media containing 1.2 mg/mL of G418 (Geneticin, Thermo Fisher, 10131027). Cells were analyzed and sorted for transfection efficiency via flow cytometry analysis using anti-human CD3 APC/Cy7–conjugated antibody and anti-human CD34 PE–conjugated antibody for TCR-expressing cells (BioLegend, 300426 at 10 μg/mL; BioLegend, 343606 at 1.25 μg/mL).

### Cell culture and ELISAs.

TCR-expressing Jurkat 76 cells were cultured overnight (20 hours) in PMA-supplemented nonselection RPMI media at a 1:1 ratio with T cell–depleted PBMCs derived either from the participant’s donor or from a third-party healthy donor. Cocultures were performed as distinct triplicates (*n* = 3). Coculture supernatant was collected following completion of culture and analyzed for IL-2 using the ELISA MAX Deluxe Set for human IL-2 (BioLegend, 431804) at a dilution of 1:5.

### Data availability.

Genomics data are available via the NCBI BioProject (PRJNA974568). Values for all data points found in graphs can be found in the supporting data values file.

### Statistics.

Statistical analyses, including *t* tests, 1-way ANOVA analyses, and linear regressions, were done using GraphPad Prism, version 9.3.1. Significance was calculated, adjusting for multiple comparisons, at *P* < 0.05. Differential gene expression analyses were performed using Seurat (version 4.1.0) function FindAllMarkers to identify significantly expressed genes at *P* < 0.05.

### Study approval.

All participants were independently enrolled in this mechanistic rejection study approved by the University of Cincinnati Institutional Review Board (IRB 2017-4696, 2019-0469). Members of one group of patients were simultaneously enrolled in the CIRRUS I trial (ClinicalTrials.gov NCT03663335). Enrollment occurred at two Cincinnati, Ohio hospitals (University of Cincinnati Medical Center and The Christ Hospital) upon scheduling of a for-cause rBx. All participants provided written, informed consent before study procedures occurred, with continuous consent ensured throughout participation.

## Author contributions

TS, ARB, BMB, ESW, and DAH designed research studies. TS, ARB, CMCR, PCC, GIG, SGF, and JA conducted experiments. ARB, RRA, ARS, and ESW recruited participants. TS, ARB, JTC, KR, PCC, GIG, JA, CMC, DAA, JSR, CHR, GW, BMB, ESW, and DAH analyzed data. BMB provided reagents. TS, ARB, JTC, JSR, CHR, GW, BMB, ESW, and DAH wrote and edited the manuscript.

## Supplementary Material

Supplemental data

Supplemental tables 1-7

Supporting data values

## Figures and Tables

**Table 2:**
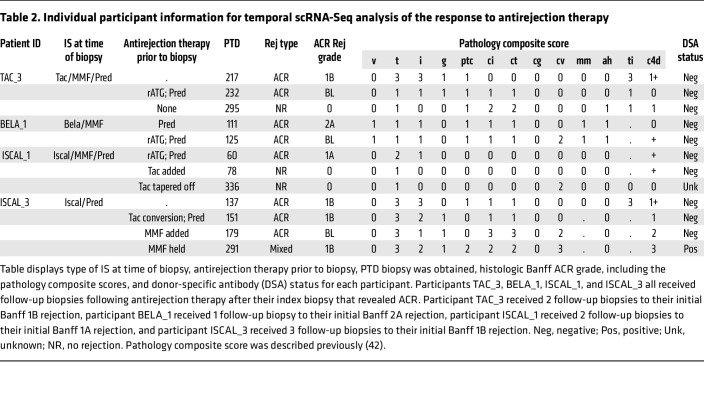
Individual participant information for temporal scRNA-Seq analysis of the response to antirejection therapy

**Table 1:**
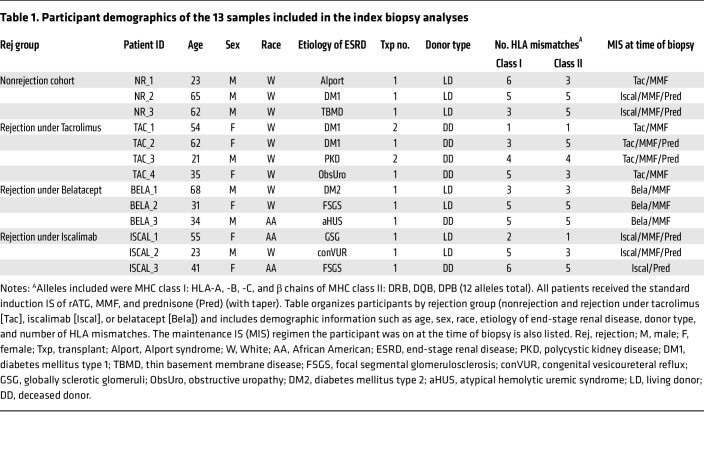
Participant demographics of the 13 samples included in the index biopsy analyses

## Version 1 05/25/2023

In-Press Preview

## Version 2 07/17/2023

Electronic publication
