# Supplementary material for: Single-cell transcriptomic analysis of renal allograft rejection reveals insights into intragraft TCR clonality
Source: J Clin Invest. 2023 Jul 17;133(14):e170191. doi: 10.1172/JCI170191 (PMC10348771; doi:10.1172/JCI170191)
Supplement: Supplemental data [file jci-133-170191-s119.pdf]

A

Rej Group	Patient ID	Total # MM	Class I HLA	A	A	B	B	C	C	# Class I MM	Class II HLA	DRB	DRB	DQB	DQB	DPB	DPB	# Class II MM
Rejection under Tacrolimus	TAC_1	2	Recipient	A02	-	B62	-	C10	-	1	Recipient	DR04	DR11	DQ07	DQ08	DP02:01	DP04:01	1
			Donor	A02	-	B60	-	C10	-		Donor	DR04	DR11	DQ07	DQ08	DP16:01	DP04:01	
	TAC_2	8	Recipient	A02	A29	B08	B45	C06	C07	3	Recipient	DR04	DR17	DQ02	DQ07	DP04:01	DP15:01	5
			Donor	A02	-	B13	B62	C06	C10		Donor	DR01	DR09	DQ05	DQ09	DP04:01	DP02	
	TAC_3	8	Recipient	A03	A29	B44	B72	C02	C16	4	Recipient	DR04	DR07	DQ02	DQ07	DP04:01	DP10:01	4
			Donor	A01	A11	B35	-	C04	-		Donor	DR04	DR01	DQ05	DQ07	DP02:01	DP03:01	
	TAC_4	8	Recipient	A24	A32	B25	B60	C04	C10	5	Recipient	DR08	DR11	DQ04	DQ07	DP03:01	DP06:01	3
			Donor	A02	-	B08	B50	C06	C07		Donor	DR08	DR04	DQ08	DQ07	DP04:01	-	
Rejection under Belatacept	BELA_1	6	Recipient	A03	-	B07	B62	C07	C10	3	Recipient	DR01	DR15	DQ05	DQ06	DP04:01	DP04:02	3
			Donor	A02	-	B08	B60	C07	C10		Donor	DR13	DR17	DQ02	DQ06	DP04:01	DP04:02	
	BELA_2	10	Recipient	A02	A32	B60	B64	C08	C10	5	Recipient	DR04	DR13	DQ06	DQ07	DP03:01	DP04:01	5
			Donor	A01	-	B08	B44	C07	C16		Donor	DR07	DR17	DQ02	-	DP01:01	DP04:02	
	BELA_3	10	Recipient	A03	A30	B56	B57	C01	C18	5	Recipient	DR03:02	DR10:01	DQ04	DQ05	DP01:01	DP17:01	5
			Donor	A02	-	B07	B61	C02	C07		Donor	DR07	DR08	DQ04	DQ02	DP02:01	DP04:01	
Rejection under Iscalimab	ISCAL_1	3	Recipient	A30	A68	B53	B65	C04	C08	2	Recipient	DR15	DR17	DQ02	DQ06	DP02:01	DP17:01	1
			Donor	A30	A32	B64	B65	-	C08		Donor	DR15	DR07	DQ02	DQ06	DP02:01	-	
	ISCAL_2	8	Recipient	A03	A68	B07	B37	C06	C07	5	Recipient	DR13	DR15	DQ06	-	DP01:01	DP04:01	3
			Donor	A03	A02	B35	B51	C04	C14		Donor	DR01	-	DQ05	-	DP15:01	DP04:01	
	ISCAL_3	11	Recipient	A23	A24	B14	B81	C08	C08	6	Recipient	DR11	DR12	DQ05:01	DQ06:02	DP105:01	-	5
			Donor	A01	A26	B44	B57	C06	C16		Donor	DR07	-	DQ02	DQ09	DP11:01	DP13:01	

B

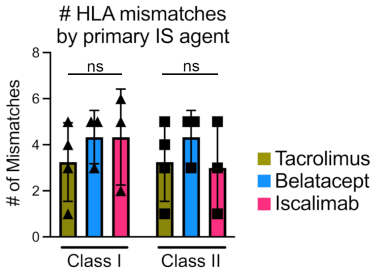

Supplemental Figure 1. Participant-donor HLA typing. (A) 2-digit HLA typing is listed for each participant for class I HLAs (left) and class II HLAs (right). Numbers of mismatches ranger from 2 to 11 total mismatched HLAs. (B) Bar graphs compare total number of class I or class II mismatches between rejection groups (tacrolimus [mustard, n=4], belatacept [blue, n=3], iscalimab [pink, n=3]) [one-way ANOVA, ns = not significant, p>0.05].

A

Rej Group	ID	IS at Time of Biopsy	PTD	Rej Type	ACR Rej Grade	Pathology Composite Score													DSA Status
						v	t	i	g	ptc	ci	ct	og	cv	mm	ah	ti	c4d	
Rejection under Tacrolimus	TAC_1	Tac/MMF	14	ACR	1A	0	1	2	2	2	1	1	0	1	0	0	.	+	Neg
	TAC_2	Tac/MMF/Pred	1963	Mixed	1A	0	2	2	1	2	2	2	1	1	1	2	3	1+	Pos (II)
	TAC_3	Tac/MMF/Pred	217	ACR	1B	0	3	3	1	1	0	0	0	0	0	0	3	1+	Neg
	TAC_4	Tac/MMF	809	ACR	1B	0	3	3	0	2	1	1	0	1	1	0	3	0	Neg
Rejection under Belatacept	BELA_1	Bela/MMF	111	ACR	2A	1	1	1	0	1	1	1	0	0	1	1	.	0	Neg
	BELA_2	Bela/MMF	1532	ACR	1B	0	3	2	0	1	1	1	0	1	0	0	2	1+	Pos (II)*
	BELA_3	Bela/MMF	172	ACR	2A	1	2	2	0	1	1	1	0	0	0	0	2	0	Neg
Rejection under Iscalimab	ISCAL_1	Iscal/MMF/Pred	60	ACR	1A	0	2	1	0	0	0	0	0	0	0	0	.	+	Neg
	ISCAL_2	Iscal/MMF/Pred	14	ACR	1A	0	1	2	0	0	0	0	0	0	0	0	2	-	Neg
	ISCAL_3	Iscal/Pred	137	ACR	1B	0	3	3	0	1	1	1	0	0	0	0	3	1+	Neg

B

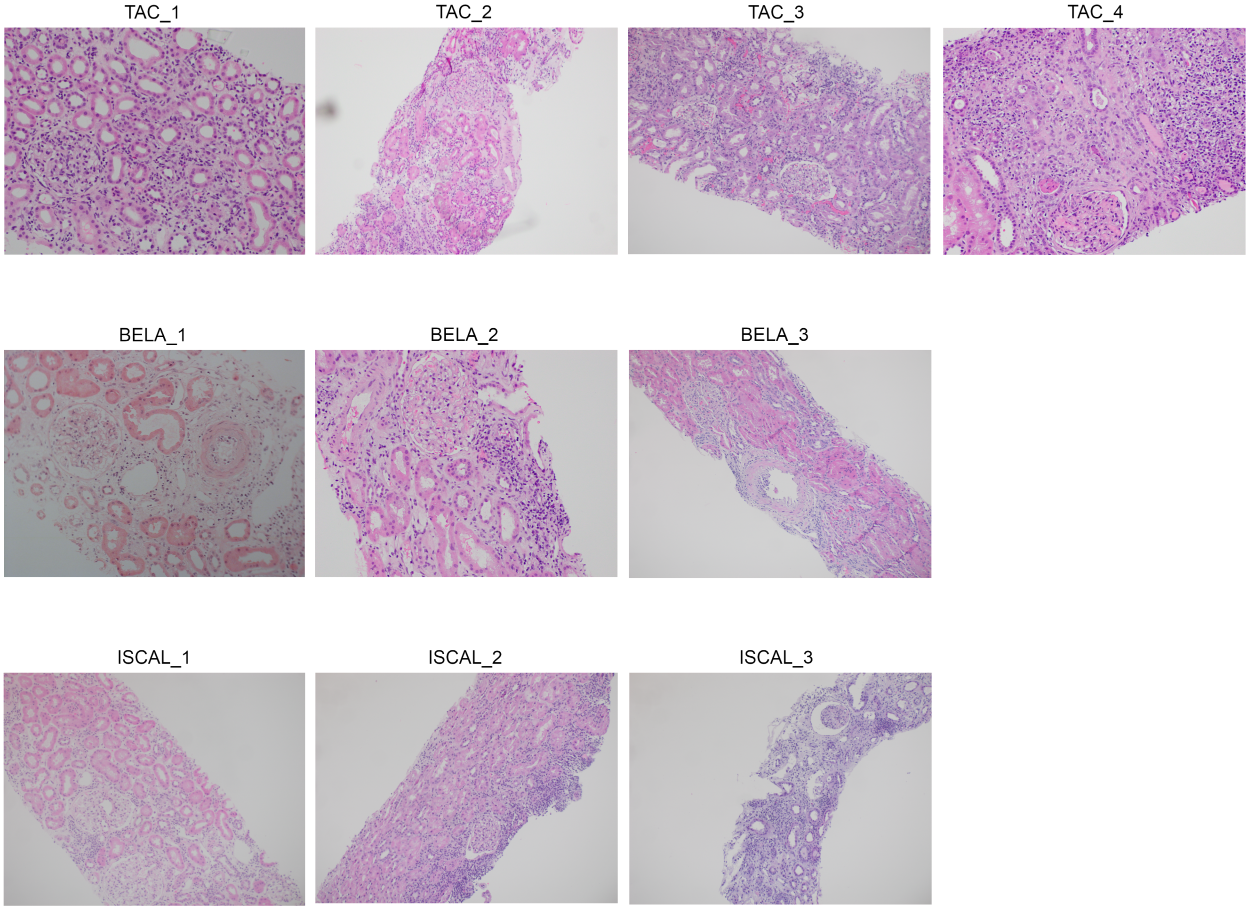

Supplemental Figure 2. Clinical and pathological characteristics of index rejection biopsies. (A) Of the 10 index biopsies obtained from participants undergoing rejection, there was an even spread across Banff rejection grades, including Banff 1A, 1B, and 2A rejections. Pathology composite scores and DSA status are provided for each sample. (B) Representative histological images taken at 10x magnification of each index biopsy sample reflect their pathological composite scores and Banff rejection grades.

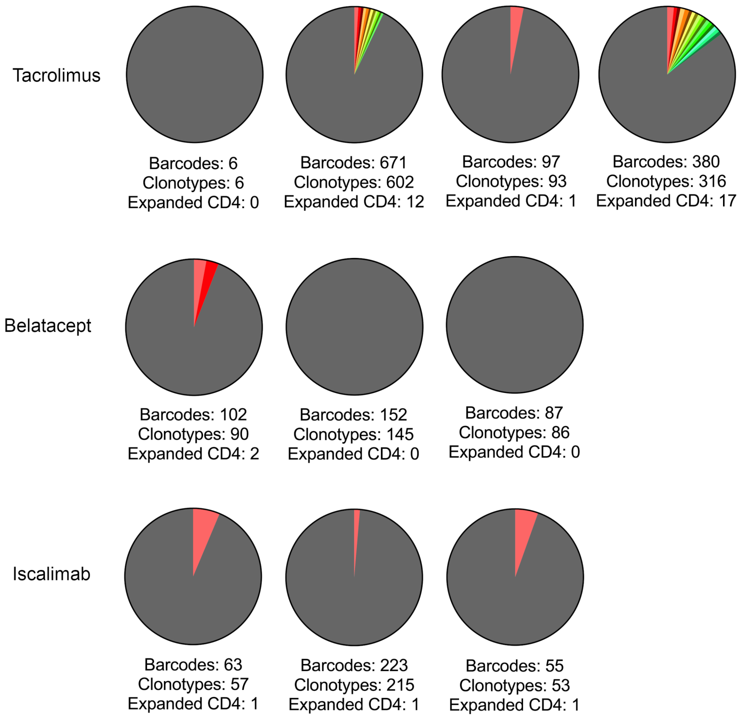

Supplemental Figure 3. Clonal analysis of CD4+ T cells from index biopsies. Pie charts display number and frequency of expanded CD4 clonotypes ($CD4_{EXP}$) found in the biopsy during rejection by participant sample, based on their unique CDR3αβ sequences. Expanded clonotypes are defined as having >2 cells with identical CDR3αβ sequences. Different colors represent individual expanded clonotypes (gray area represents unexpanded clonotypes) and the size of the colored area represents the relative size of the $CD4_{EXP}$.

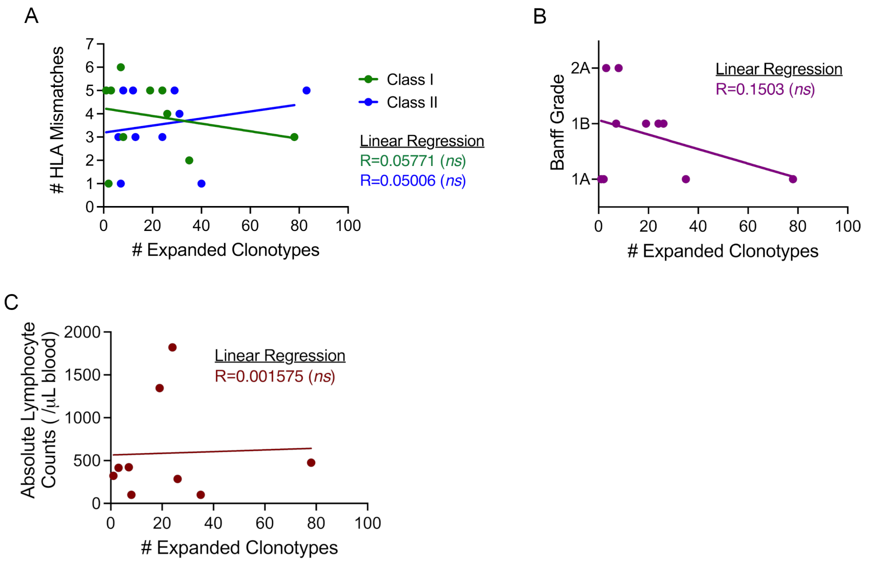

Supplemental Figure 4. No correlation between numbers of CD8EXP and numbers of HLA mismatches, Banff rejection grade, or absolute lymphocyte count. (A-C) Simple linear regression analysis [ns = not significant, $p>0.05$] were performed on number of CD8EXP against number of (A) HLA mismatches, (B) Banff rejection grade, and (C) absolute lymphocyte count (ALC).
